# Comment on Rodríguez-Cortés et al. Individual Circadian Preference, Eating Disorders and Obesity in Children and Adolescents: A Dangerous Liaison? A Systematic Review and a Meta-Analysis. *Children* 2022, *9*, 167

**DOI:** 10.3390/children11111335

**Published:** 2024-10-31

**Authors:** José Francisco López-Gil, Juan Ramón Barrada

**Affiliations:** 1 One Health Research Group, Universidad de Las Américas, Quito 170124, Ecuador; 2 Department of Psychology and Sociology, University of Zaragoza, 44003 Teruel, Spain; barrada@unizar.es

**Keywords:** chronobiology, evening chronotype, morning chronotype, disordered eating, other specified feeding or eating disorders, food addiction, night eating syndrome, pediatric health

## Abstract

This commentary critically examines the article by Rodríguez-Cortés et al. on the links between circadian preferences, eating disorders, and obesity in pediatric populations, emphasizing the association between evening chronotypes and disordered eating behaviors. Key findings highlight the increased prevalence of food addiction (FA) and night eating syndrome (NES) among evening-oriented children and adolescents, though the article’s reliance on studies with adult samples limits the generalizability of its conclusions. Additionally, the ambiguous classification of FA and NES within broader eating disorder frameworks necessitates further investigation to delineate these behaviors from traditional disorders such as anorexia and bulimia nervosa. This commentary advocates for future research focusing on pediatric populations to explore the intersection of circadian misalignment with emotional regulation and environmental factors, aiming to develop tailored preventative strategies that incorporate chronobiological and lifestyle modifications.

## 1. Introduction

This commentary is intended to express appreciation for the insightful article “Individual Circadian Preference, Eating Disorders, and Obesity in Children and Adolescents: A Dangerous Liaison? A Systematic Review and Meta-Analysis” by Rodríguez-Cortés et al. [1], published in *Children*. The article provides a comprehensive review of how circadian preferences, particularly being an evening chronotype, are linked to various eating disorders and obesity in young populations. While the article contributes valuable findings to the field, there are several opportunities to build upon its conclusions and expand the conversation, especially in the context of child and adolescent health.

## 2. Evening Chronotype and Its Relationship with Eating Behavior

Rodríguez-Cortés et al. [1] shed light on the relationship between being an evening chronotype and unhealthy eating behaviors, an issue that has gained significant attention in recent years. Adolescents and children who favor later sleep times tend to exhibit a higher body mass index (BMI) and are more likely to consume junk food, often associated with disordered eating patterns such as food addiction (FA) and night eating syndrome (NES). These findings align with broader research suggesting that being an evening chronotype may lead to circadian misalignment, where the biological clock is out of sync with social and environmental demands, further exacerbating unhealthy lifestyle choices [2].

However, while the review by Rodríguez-Cortés et al. [1] highlights this association, it also leaves certain gaps, particularly the lack of evidence linking being an evening chronotype with traditional eating disorders such as anorexia nervosa or bulimia nervosa in pediatric populations. This gap highlights the need for future research to explore the possible connections between being an evening chronotype and these specific disorders, as current studies tend to focus predominantly on binge eating disorder. Additionally, it remains unclear whether circadian rhythm disruptions in adolescents contribute differently to disordered eating behaviors compared to adults, an area that deserves more targeted investigation [3].

## 3. Expanding the Scope of Food Addiction and Night Eating Syndrome

While FA and NES are central to Rodríguez-Cortés et al.’s analysis [1], both conditions remain somewhat ambiguous in their classification within the broader spectrum of eating disorders. FA, for example, is characterized by intense cravings and loss of control-related overeating habits but is not currently recognized as a formal eating disorder in major diagnostic frameworks like *The Diagnostic and Statistical Manual of Mental Disorders, Fifth Edition* (DSM-5-TR) [4]. NES, involving night-time eating and disrupted sleep, is similarly listed under “Other Specified Feeding or Eating Disorders”, further complicating its status [5]. Additionally, it should be noted that NES (classified as an “other specified feeding or eating disorder”) must be distinguished from nocturnal sleep-related eating disorder (NSRED), which is a combination of a parasomnia and an eating disorder. Although these conditions appear similar, they require different clinical approaches [6]. In NES, eating episodes occur with full awareness, whereas in NSRED, there is a range of awareness during nocturnal eating episodes, ranging from no awareness to full awareness, although individuals may not be able to avoid or stop eating [4]. This distinction is crucial for appropriate treatment.

It is essential for parents, guardians, and healthcare providers to be aware of not only diagnosed eating disorders but also the signs of disordered eating. Disordered eating encompasses behaviors such as dieting for weight loss, episodes of overeating, self-induced vomiting, excessive exercise, or the use of laxatives or diuretics [7]. It is crucial to differentiate disordered eating from formal eating disorders [8], especially in children and adolescents. Disordered eating often refers to a range of eating behaviors that do not meet the full criteria for an eating disorder and therefore cannot be officially classified as such [9]. A previous systematic review and meta-analysis revealed that around about one in five children and adolescents engage in disordered eating behaviors, with higher prevalence observed in girls, older adolescents, and those with a higher BMI [10]. While these behaviors may not qualify for a clinical diagnosis, they can still lead to adverse outcomes related to eating disorders and obesity in adolescents [11]. Despite their often-overlooked impact on health, disordered eating habits should be carefully monitored, as they have the potential to progress into full-blown eating disorders [7].

One area that could benefit from additional research is how FA and NES intersect with mental health and emotional regulation, particularly in children and adolescents who may be more vulnerable to these conditions due to developmental and hormonal changes. For instance, the role of stress, anxiety, and depression in exacerbating FA or NES behaviors has been observed in adults, but these mechanisms are not well understood in younger populations. The developmental plasticity of the adolescent brain may make it more susceptible to the long-term impacts of circadian misalignment, potentially making conditions like FA and NES more persistent if not properly addressed [12,13].

Moreover, it is important to consider the influence of environmental factors, such as increased screen time, irregular meal patterns, and heightened academic or social pressures, which may further contribute to the development of FA or NES in this age group. Rodríguez-Cortés et al.’s review would have benefitted from undertaking a more detailed exploration of these factors, particularly how modern lifestyle behaviors compound the relationship between chronotype and eating disorders [14].

## 4. Limitations in Representing Younger Populations and the Lack of Significant Correlation

On the other hand, in their analysis of the relationship between chronotype and FA, Rodríguez-Cortés et al. [1] only included one study; for the relationship between chronotype and NES, they included two studies. Out of these three studies, participants in two were university students (Kandeger et al. [15]: M*_age_* = 21.1 years; Najem et al. [16]: M*_age_* = 20.2 years). Therefore, most of the samples involved were not children or adolescents. Surprisingly, Rodríguez-Cortés et al. [1], when describing the results of Kandeger et al. [15], did not reported that the correlation between chronotype and Eating Attitudes Test-26 (EAT-26) scores (a common screening measure of eating disorders) was –0.03 and non-statistically significant.

## 5. Integrating a Holistic Approach to Preventative Measures

Given the growing body of evidence linking circadian rhythms with eating disorders and obesity, it is clear that future preventative strategies should adopt a more holistic approach. Rather than focusing solely on dietary interventions, it may be beneficial to incorporate circadian-aligned lifestyle modifications. For instance, promoting regular sleep patterns that align with natural light–dark cycles could help to mitigate the negative effects of being an evening chronotype on eating behaviors. Additionally, interventions that emphasize stress management, emotional regulation, and physical activity could further strengthen the prevention and treatment of these disorders [7].

Furthermore, there is increasing recognition that personalized interventions tailored to an individual’s circadian typology (whether morning, intermediate, or evening type) may yield better outcomes. This approach could be particularly relevant for children and adolescents, whose biological clocks are naturally more flexible. By incorporating chronobiological principles into treatment plans, clinicians and researchers may be able to offer more precise, targeted therapies that resonate with an individual’s unique circadian preferences and needs.

## 6. Additional Information on the Quality of the Review and Risk of Bias

The systematic review by Rodríguez-Cortés et al. addresses an important topic—the association between circadian preferences, eating behaviors, and obesity in children and adolescents. However, there are notable limitations in terms of quality and risk of bias. The application of the A Risk of Bias Assessment Tool for Systematic Reviews (ROBIS) [17] reveals concerns regarding the inclusion of non-target populations, particularly studies that focused on university-aged participants, thus weakening the generalizability of the findings to children and adolescents (Table S1). Additionally, the review focuses primarily on an atypical eating disorder (i.e., NES) and a disordered eating (i.e., FA), while no studies on more common eating disorders, such as anorexia nervosa and bulimia nervosa, are identified, which further narrows the scope of the review.

Moreover, the supplementary materials provided by Rodríguez-Cortés et al. [1] do not include the complete quality assessment of the three studies included in the systematic review or the meta-analysis, which is a crucial omission. This gap limits transparency and raises concerns about the robustness of the conclusions, particularly in the absence of sensitivity analyses or funnel plots to assess publication bias. As a result, while the findings are informative, they must be interpreted cautiously due to these limitations in study inclusion and the incomplete quality assessment of the meta-analyzed studies.

## 7. Conclusions

Rodríguez-Cortés et al.’s [1] review provides important insights into the relationship between chronotype, eating disorders, and obesity, but limitations arise from the use of adult samples rather than children or adolescents, making the generalization of findings to younger populations problematic. Additionally, the inclusion of atypical conditions like FA and NES complicates the narrative, as these behaviors differ from traditional eating disorders and require independent investigation. Future research should focus specifically on pediatric populations and distinguish between these conditions to develop more precise prevention and treatment strategies.

## Supplementary Materials

The following supporting information can be downloaded at: https://www.mdpi.com/article/10.3390/children11111335/s1, Table S1: Risk of bias assessment using the Risk of Bias in Systematic Reviews (ROBIS) Tool.
