# Supplementary material for: Comment on Rodríguez-Cortés et al. Individual Circadian Preference, Eating Disorders and Obesity in Children and Adolescents: A Dangerous Liaison? A Systematic Review and a Meta-Analysis. Children 2022, 9, 167
Source: Children (Basel). 2024 Oct 31;11(11):1335. doi: 10.3390/children11111335 (PMC11592937; doi:10.3390/children11111335)
Supplement: Supplementary file 1 [file children-11-01335-s001.zip › children-3237839-supplementary.pdf]

**Table S1.** Risk of bias assessment using the Risk of Bias in Systematic Reviews (ROBIS) Tool.

**<u>Phase 1: Assessing Relevance (Optional)</u>**

For aetiology reviews:

| Category | Target question (e.g. overview or guideline) | Review being assessed |
|---|---|---|
| **Patients/Population(s):** | Are circadian preferences (chronotypes) associated with eating disorders and obesity in children and adolescents? | Children and adolescents (although some studies included university students, which weakens the relevance to the target population). |
| **Exposure(s) and comparator(s):** | Circadian preferences (morningness vs. eveningness) | Studies examining the association between circadian preferences and eating behaviors (focused on Food Addiction (FA), Night Eating Syndrome (NES), and obesity). |
| **Outcome(s):** | Eating disorders (FA, NES) and obesity in children and adolescents | Body Mass Index (BMI), presence of eating disorders (though FA and NES are atypical), and associations between chronotype and disordered eating behaviors. |

**- Does the question addressed by the review match the target question?**
Answer: **Yes** – The review does address the relationship between circadian preference and eating behaviors, though it misses key disorders (anorexia, bulimia, etc.).

**Phase 2: Identifying Concerns with the Review Process**

**DOMAIN 1: Study Eligibility Criteria**

**- Study eligibility criteria description:**
The review includes observational studies assessing the relationship between circadian preferences, eating disorders, and obesity in children and adolescents. However, two of the three studies addressing food addiction and night eating syndrome involved university-aged participants, raising concerns about population relevance.

**- 1.1 Did the review adhere to pre-defined objectives and eligibility criteria?**
Answer: **PY (Probably Yes)** – The review did generally adhere to its criteria but deviated slightly by including older participants.

**- 1.2 Were the eligibility criteria appropriate for the review question?**
Answer: **PN (Probably No)** – The inclusion of university-aged participants diluted the relevance for the target population.

**- 1.3 Were eligibility criteria unambiguous?**
Answer: **PY (Probably Yes)** – The eligibility criteria were clear, but the execution allowed for some older participants.

**- 1.4 Were any restrictions in eligibility criteria based on study characteristics appropriate (e.g., date, sample size, study quality, outcomes measured)?**
Answer: **Y (Yes)** – Restrictions based on study quality and design were appropriate, though the lack of a strict age restriction was a limitation.

**- 1.5 Were any restrictions in eligibility criteria based on sources of information appropriate (e.g., publication status or format, language, availability of data)?**
Answer: **Y (Yes)** – No inappropriate restrictions were imposed.

**- Concerns regarding specification of study eligibility criteria**
Answer: **High**.

**Rationale for concern:** The inclusion of university-aged participants affected the applicability of results to children and adolescents.

**DOMAIN 2: Identification and Selection of Studies**

**- Methods of study identification and selection description:**
The review searched six major databases and included observational studies. However, it missed key studies on typical eating disorders (e.g., anorexia, bulimia, binge eating disorder) in children and adolescents.

**- 2.1 Did the search include an appropriate range of databases/electronic sources for published and unpublished reports?**
Answer: **Y (Yes) –** A wide range of databases were used, but relevant studies were still overlooked.

**- 2.2 Were methods additional to database searching used to identify relevant reports?**
Answer: **N (No) –** There were no additional methods like hand searching or contacting authors.

**- 2.3 Were the terms and structure of the search strategy likely to retrieve as many eligible studies as possible?**
Answer: **PN (Probably No) –** Despite the range of databases, relevant studies were missed, particularly on typical eating disorders.

**- 2.4 Were restrictions based on date, publication format, or language appropriate?**
Answer: **Y (Yes) –** No inappropriate restrictions were applied.

**- 2.5 Were efforts made to minimize error in the selection of studies?**
Answer: **PY (Probably Yes) –** Two reviewers independently screened the articles.

**- Concerns regarding methods used to identify and/or select studies**
Answer: Moderate.

**Rationale for concern:** Despite a comprehensive search, important studies were missed, especially those on common eating disorders in children.

**DOMAIN 3: Data Collection and Study Appraisal**

**- Methods of data collection and risk of bias appraisal description:**
Data collection adhered to Cochrane guidelines. However, the focus on FA and NES, which are atypical behaviors not classified as typical eating disorders, limits the review's relevance. Risk of bias was evaluated appropriately using standard criteria.

**- 3.1 Were efforts made to minimize error in data collection?**
Answer: **Y (Yes)** – Standardized protocols were followed.

**- 3.2 Were sufficient study characteristics available for both review authors and readers to be able to interpret the results?**
Answer: **Y (Yes)** – Study characteristics were provided.

**- 3.3 Were all relevant study results collected for use in the synthesis?**
Answer: **PN (Probably No)** – Only a few studies focused on FA and NES, excluding typical eating disorders from the synthesis.

**- 3.4 Was risk of bias (or methodological quality) formally assessed using appropriate criteria?**
Answer: **Y (Yes)** – Risk of bias was assessed using appropriate criteria.

**- 3.5 Were efforts made to minimize error in risk of bias assessment?**
Answer: **PY (Probably Yes)** – Two reviewers were involved, but the selection of studies with non-target age groups affected the bias assessment.

**- Concerns regarding methods used to collect data and appraise studies**
Answer: **Moderate**.

**Rationale for concern:** The reliance on atypical behaviors (FA, NES) and the inclusion of older participants weakens the review's applicability to children and adolescents.

**DOMAIN 4: Synthesis and Findings**

**- Synthesis methods description:**
The review provided a narrative synthesis and a quantitative meta-analysis based on three studies. However, the synthesis excluded key studies on anorexia, bulimia, and binge eating disorder in children. Additionally, heterogeneity between studies was not fully addressed.

**- 4.1 Did the synthesis include all studies that it should?**
Answer: **N (No) –** The synthesis included studies with university-aged participants, which are not representative of the target population (children and adolescents).

**- 4.2 Were all pre-defined analyses reported or departures explained?**
Answer: **Y (Yes) –** All pre-defined analyses were reported.

**- 4.3 Was the synthesis appropriate given the nature and similarity in the research questions, study designs, and outcomes across included studies?**
Answer: **PN (Probably No) –** There was significant variation in study design and population (some studies included young adults).

**- 4.4 Was between-study variation (heterogeneity) minimal or addressed in the synthesis?**
Answer: **Y (Yes) –** There was no significant heterogeneity ($I^2 = 0\%$). However, this fact was not explicitly mentioned in the discussion, which could have reinforced the consistency of the results. While not strictly necessary, acknowledging the lack of heterogeneity would have provided additional clarity.

**- 4.5 Were the findings robust, e.g., as demonstrated through funnel plot or sensitivity analyses?**
Answer: **NI (No Information) –** Neither a funnel plot nor a sensitivity analysis was reported, possibly due to the limited number of studies included.

**- 4.6 Were biases in primary studies minimal or addressed in the synthesis?**
Answer: **PN (Probably No) –** Biases related to population age were not sufficiently addressed.

**- Concerns regarding the synthesis and findings**
Answer: **High**.

**Rationale for concern:** The limited number of included studies and the exclusion of key eating disorders weakened the findings.

**Phase 3: Judging Risk of Bias**

*- Summarize the concerns identified during the Phase 2 assessment:*

**- Domain 1: High concern** due to the inclusion of older participants not fitting the target population.

**- Domain 2: Moderate concern** due to missed studies on typical eating disorders.

**- Domain 3: Moderate concern** due to the focus on atypical eating behaviors and the inclusion of older participants.

**- Domain 4: High concern** due to limited synthesis and the exclusion of key disorders.

**- Risk of bias in the review:**
Answer: **High**.

**Rationale for risk:** The review's conclusions are undermined by the inclusion of non-target populations (university-aged participants) and the focus on atypical eating behaviors (i.e., NES) or disordered eating (i.e., FA). Furthermore, the absence of studies addressing eating disorders (such as anorexia nervosa, bulimia nervosa, or binge eating disorder) in children and adolescents limits the comprehensiveness of the review. While this absence reflects a gap in the current literature rather than an oversight by the authors, it significantly weakens the strength of the conclusions drawn about the relationship between circadian preferences and disordered eating in this population.
